# Biomedical research in Alzheimer’s disease in a Latin American developing country: challenges in the informed consent process

**DOI:** 10.1093/braincomms/fcag011

**Published:** 2026-01-14

**Authors:** Natalia Salvadores

**Affiliations:** Neurodegenerative Diseases Laboratory, Center for Biomedicine, Universidad Mayor, Temuco 4780000, Chile; Comité Ético Científico, Universidad Mayor, Temuco 4780000, Chile

**Keywords:** Alzheimer’s disease, biomedical research, Latin America, informed consent process

## Abstract

Alzheimer’s disease (AD) is a significant global health issue, impacting 50 million individuals with dementia worldwide, a number projected to triple by 2050. Although research has been conducted for decades, there is no effective prevention, treatment, or early diagnostic method for AD. Research on AD in Latin America (LatAm) faces unique challenges compared to developed countries due to socioeconomic, cultural, and infrastructural factors. While the prevalence of dementia is rapidly increasing in LatAm due to demographic shifts, the region is underrepresented in research, diagnostics and care. The informed consent process, a critical aspect of research, becomes particularly complex with individuals who have cognitive impairments. It requires a balance between protecting vulnerable individuals and advancing research for their benefit. By developing and implementing best practices, ethical research can be conducted with this population, ensuring they receive appropriate care. This review provides an update on informed consent for AD research in Chile.

## Teaser

Salvadores reviews the challenges of the informed consent process in Alzheimer’s research, highlighting the ethical complexities of working with cognitively impaired participants and the regional disparities affecting Latin America. The review concludes that implementing tailored best practices can protect participants and promote equitable and ethical research development across the region.

## Graphical Abstract

**Graphical Abstract:**
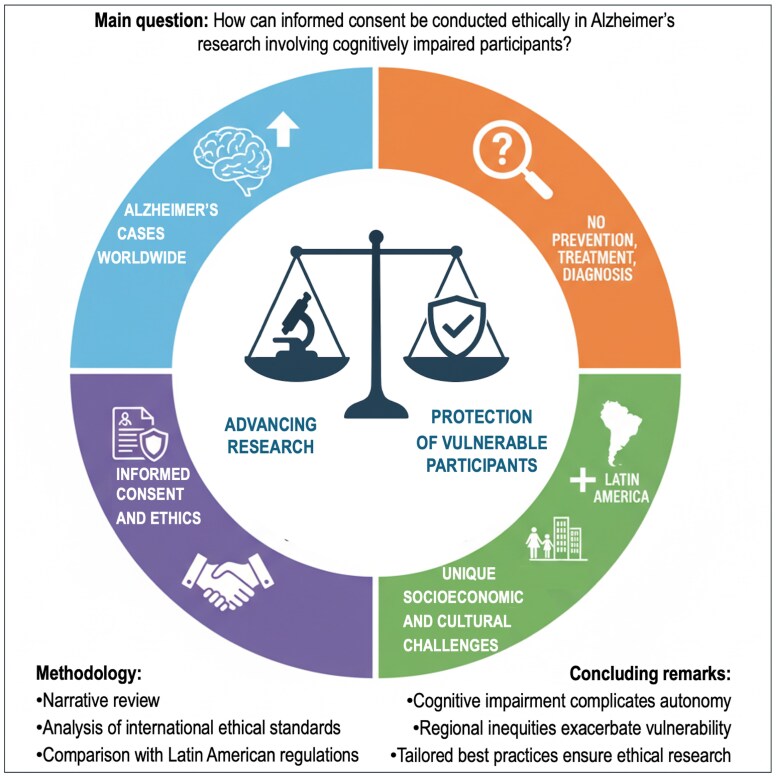

## Alzheimer’s disease overview

Alzheimer’s disease (AD) is a neurodegenerative disorder and the most common cause of dementia, characterized by a decline in cognitive and functional abilities.^1-3^ The disease has an enormous global impact with an estimated 50 million people affected, a number expected to triple by 2050. AD places a significant burden on individuals, families and the economy.^1,4^

The primary hallmarks of AD include the presence of amyloid plaques and neurofibrillary tangles in the brain. Amyloid plaques are formed by the accumulation of beta-amyloid (Aβ) peptide, while neurofibrillary tangles consist of hyperphosphorylated tau protein.^1^ The amyloid hypothesis suggests that the accumulation of Aβ is the primary event that initiates a cascade of neurodegenerative processes. This cascade includes synaptic dysfunction, neuronal loss and neuroinflammation.^3^ The disease is now understood to be a continuum, progressing from a preclinical phase with no symptoms to mild cognitive impairment, and eventually to overt dementia.^1,5^

Both modifiable and non-modifiable factors influence the risk of AD. Non-modifiable factors include increasing age and genetic predisposition.^2^ Modifiable risk factors include head injuries, infections, cardiovascular risk factors and obesity, among others.^1,2^

Diagnosis of AD is based on clinical criteria and can be supported by biomarkers. Diagnostic criteria have evolved over time, with the most recent guidelines recognizing the importance of preclinical stages.^5^

Biomarkers include brain imaging markers and cerebrospinal fluid markers. While these biomarkers are valuable, they are not globally accessible due to high costs.^6-9^ Biological markers of Aβ and tau are more commonly standardized in Europe and the USA but are unavailable in Latin American (LatAm) countries for diagnostic purposes.^6,9^ Currently, there is no cure for AD. Existing treatments focus on managing symptoms but do not slow or stop the underlying disease progression. Cholinesterase inhibitors and NMDA receptor antagonists are approved for symptomatic treatment. These drugs can temporarily improve cognition and daily functioning.^10^ Disease-modifying therapies are developing, targeting mechanisms such as Aβ aggregation and tau pathology. In this line, immunotherapy with monoclonal antibodies has shown promise in removing Aβ plaques and slowing cognitive decline, which led to the recent approval of lecanemab and donanemab by the Food and Drug Administration of the USA and the approval of lecanemab by the European Medicines Agency (EMA).^11-13^

## AD research in the context of Latin America

Biomedical research plays a crucial role in advancing our understanding of AD and therefore, developing effective diagnostic and therapeutic strategies. The complexity of AD, with its multifaceted pathology and diverse risk factors, requires rigorous investigation across various scientific disciplines. By developing reliable biomarkers, innovative therapies and preventative approaches, the scientific community is working on strategies aimed at improving the lives of those affected by AD. Continued focus on basic science, drug development and clinical trials is essential for translating research findings into effective interventions for this devastating disease.

LatAm is experiencing a faster increase in AD prevalence compared to Europe and North America, with an overall dementia prevalence of 7.1%.^14-16^ In the context of AD research, LatAm faces unique challenges due to a combination of factors that contribute to disparities in the field. These disparities manifest in several ways. A key challenge in AD research is the underrepresentation of diverse populations, particularly those from LatAm, in studies, which leads to results that may not be generalizable to all groups.^6,17^ This issue arises from two main factors. First, a large portion of AD research, especially studies that compare different ethnic groups, is conducted in developed countries, such as the USA and European nations.^9,18^ This over-representation limits the understanding of how AD may manifest and progress in other groups. For example, a study showed that as of 2018, most genome-wide association studies across human diseases had been conducted in European individuals, and only 1% involved LatAm subjects.^19^ Second, when studies do include minority populations, including those from LatAm, these individuals often come from very different socioeconomic backgrounds compared to the majority group.^18^ This means that factors like educational attainment, income, access to healthcare and exposure to environmental risk factors may vary widely. Such differences can influence the validity of the studies.

Low socioeconomic and educational levels in the region contribute to a major increase in the prevalence of dementia.^20,21^ Moreover, cultural and linguistic barriers also affect the accurate diagnosis of dementia. There are over 400 different indigenous groups within LatAm, and many people have low levels of literacy and education. Furthermore, the lack of culturally appropriate cognitive assessment tools can lead to biased results when applied to individuals from different cultural backgrounds.^14,16,18,22^

**Figure 1:**
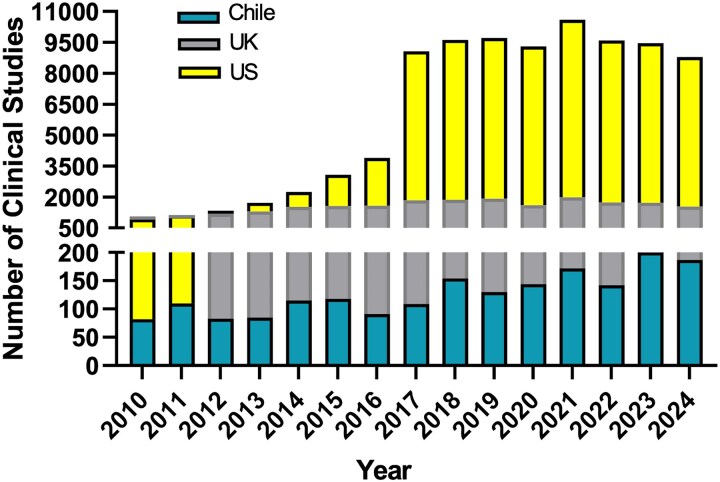
**Number of clinical trials in Chile, the UK and the USA**. The graph shows the total number of clinical studies conducted in Chile (*n* = 1922), the UK (*n* = 21 579) and the USA (*n* = 90 470), between 2010 and 2024, that were registered in ClinicalTrials.gov.

In terms of infrastructure and resources, many LatAm countries have minimal mental health facilities and lack specific mental health policies or dedicated budgets for dementia care. Health facilities are often concentrated in large cities, leading to shortages of specialists in rural areas.^9,14,22^ Additionally, specialized services are frequently only covered by private health insurance, which limits access for many individuals. There is also a lack of primary healthcare programmes to address diagnosis and ensure timely referrals, as well as limited information on the referral process when dementia is suspected. Furthermore, access to advanced neuroimaging and biomarker technologies remains limited.^6,9^ The restricted availability of funding is a critical barrier to implementing biomarker research and clinical trials. A comparison of the number of clinical studies performed in Chile, the UK and the USA between 2010 and 2024 shows a moderate overall increase in Chile, roughly doubling across the period, while both the UK and the USA exhibit much larger proportional growth. In several key years, the UK conducted more than 10 times the number of trials seen in Chile, and the USA expanded even more sharply, reaching levels dozens of times higher. Chile’s pattern is relatively stable with small fluctuations, whereas the UK shows steady growth, and the USA displays a steep surge after the mid-2010s followed by stabilization at very high levels. Overall, Chile’s activity grows but remains modest compared with the massive scale and steeper trajectories observed in the UK and USA (Fig. 1).

Despite the challenges described above, LatAm offers unique opportunities for research. The region is home to diverse populations with varied genetic ancestries and environmental exposures, which could provide valuable insights into the aetiology of AD.

## Relevance of informed consent in biomedical research

Informed consent is a critical element in biomedical research, arising from a history of abuses in both clinical and research settings.^23^ It is rooted in the ethical principles of respecting individual autonomy and ensuring that participation in research is voluntary. In biomedical research, the Nuremberg Code, the Declaration of Helsinki and the Belmont Report establish fundamental international standards for the development of this document.^24-26^ The concept of informed consent has evolved due to advances in medicine and the type of data being gathered. It is not just a signed document; rather, it represents a multifaceted bridge between researchers and participants.^27^

The core of informed consent involves three key aspects: full disclosure of study information, adequate comprehension by the participant and voluntary participation.^23^ The disclosure component requires that participants are provided with all necessary details about the study, including its purpose, procedures, potential risks and benefits, alternative options and the right to ask questions and withdraw at any time. Comprehension means the information is presented in a way that is easily understandable to the study population, avoiding complex legal or technical language. Voluntariness means the decision to participate must be free of any coercion or undue influence.^23^

## Cognitive difficulties in AD and their impact on informed consent

Informed consent is particularly complex when dealing with individuals who have cognitive impairments, such as those with AD. The challenge arises from the fact that AD progressively impairs cognitive functions, including memory, reasoning, attention and language, which are essential for making informed decisions.^28^ This raises significant concerns about a person’s capacity to understand the implications of treatment or research participation and provide valid consent. While the law presumes that all individuals are capable of making decisions unless proven otherwise,^29^ the presence of cognitive impairment introduces a layer of complexity requiring careful assessment and consideration. The core of informed consent rests on the ability of an individual to receive and understand information; process this information; appreciate the situation and its consequences; weigh benefits, risks and alternatives; and ultimately, communicate a decision.^23^ These capacities can be significantly affected by AD,^1^ making the informed consent process particularly challenging.

The impact of AD on decision-making capacity is not uniform and varies based on the stage and severity of the disease. Mild cognitive impairment, which is considered a precursor to dementia,^30^ can substantially affect a person’s ability to provide informed consent, with studies indicating that a significant percentage of individuals with mild cognitive impairment may lack the capacity to make informed decisions about research participation.^31-33^ Yet, the presence of cognitive deficits does not automatically equate to an inability to make decisions. People with mild-to-moderate dementia may still retain the ability to think logically and make decisions, highlighting the need for cautious and individualized assessments. This has been supported by the finding that a substantial percentage of patients with mild-to-moderate AD can still make informed decisions regarding their treatment.^34,35^ However, these capacities may not be constant and can be influenced by various factors, including the progression of the disease, medications and even the time of day, with some individuals experiencing variations in their capacity.^36^ Furthermore, the complexity of the information and the decisions that need to be made also play a significant role. For instance, an individual with cognitive impairment may comprehend and consent to a surgical procedure but may not be able to understand the process involving anaesthesia.^37^ This indicates that capacity is not a global attribute, but is specific to the particular decision at hand, and must be assessed in that context.

The assessment of decision-making capacity in individuals with AD is crucial but not straightforward. It requires a nuanced approach, going beyond a simple cognitive test.^38^ Indeed, significant variability in clinicians’ assessments of the cognitive capacity of mild AD patients has been observed, with high rates of disagreement among their judgements.^39,40^ This discrepancy highlights the difficulty of establishing an unequivocal boundary between capacity and incapacity and emphasizes the need for careful, individualized assessments, as well as the use of validated assessment instruments. Clinicians must handle the challenge of respecting a patient’s autonomy while also ensuring actions are taken in the patient’s best interest, a balance that can be hard to strike in real-world settings. Some widely used instruments include the MacArthur Competence Assessment Tool (MacCAT-CR),^41^ the University of California, San Diego Brief Assessment of Capacity to Consent^42^ and the Mini-Mental State Examination.^43^ However, these tools have limitations. For example, the MacCAT-CR is a useful tool but requires training and up to 20 min to administer.^44^ While the MMSE is a widely used cognitive screening instrument, it does not directly measure decision-making capacity. Cognitive ability is not the only element of competence, as emotional and judgement competence should also be considered. An individual’s ability to make decisions that align with their own values and beliefs is also important. This approach suggests a need to consider the unique values and preferences of individuals when making decisions about their care and research participation.^40,45^

When an individual is deemed incapable of providing informed consent, alternative methods are required to respect the person’s self-determination to the extent possible. One such method is the use of advance directives, which are written instructions that express a person’s wishes about medical treatments in the event that they become unable to exercise self-determination.^38^ Proxy decision-making, where a family member or legal guardian makes decisions on behalf of the patient, is another alternative.^46^ In these situations, it is important to ensure that proxy decisions align as closely as possible with the individual’s values. This involves considering authenticity, which focuses on maintaining congruence between the individual’s values and the decisions that are made, even by a surrogate.^40,47^ It is worth mentioning that a significant percentage of patients with AD retain the capacity to designate a research proxy, at least in the early stages of the disease,^40^ suggesting this option should be considered more often. Researchers must also be aware of the concept of ‘process consent’, which requires researchers to continually confirm and assess the patient’s willingness to participate throughout the research process.^48^ This involves not only repeating information but assessing whether participation continues to be voluntary and allowing the individual to withdraw at any time.

To facilitate informed consent among individuals with cognitive impairments, a number of adaptations are necessary. These include simplifying consent forms, using clear and visual instructions, introducing educational approaches, providing explanations as needed and working closely with caregivers. The use of multimodal presentations such as videos, pictures and storybooks has been suggested as a way to enhance understanding.^38,45,46^ However, the most effective way to improve participant understanding might be to spend more time having direct discussions with potential study participants.^49^ In addition to this, the level of risk and burden of the research project should be carefully weighed against its potential benefits when considering whether a person is capable of consenting. The greater the risks and burdens of the study, the higher the level of decision-making ability a person must have in order to give valid consent.^44^

## Regulations in Chile on the informed consent of people with dementia

In order to establish a regulatory framework for biomedical research in humans in Chile, in 2006 the 20.120 law ‘On Scientific Research in the Human Being, Its Genome, and Prohibits Human Cloning’ was promulgated.^50^ In line with international standards, this law establishes principles and regulations regarding scientific research involving human subjects, with the aim of ensuring that such research will be conducted under ethical principles, respecting human dignity and the rights of the individuals involved. However, this law raised concerns and has been a matter of numerous critiques, including the strictness of the Article 11 regarding informed consent, which does not consider exceptions to the process.^51,52^ This moves away from what is suggested in the documents that define international benchmarks, which offer more comprehensive and precise guidelines regarding the required safeguards for research involving vulnerable populations. This includes the Declaration of Helsinki,^53^ which states that in cases where potential research subjects cannot provide informed consent such as individuals with severe mental incapacity, informed consent may be waived if the situation is addressed in the study protocol and approved by an ethics committee. Similarly, according to the Council for International Organizations of Medical Sciences (CIOMS) guidelines, research can begin without informed consent if approved by an ethics committee, but the committee must ensure that the research would not be feasible without the waiver, has significant social value and poses minimal risks to participants.^54^

In 2012, the scientific community in Chile was stunned after the promulgation of the 20.584 law^55^ that regulates the rights and duties of individuals in relation to actions linked to their healthcare.^51,52,56-58^ Specifically, the Article 28 of this law stated that: ‘No person with a mental or intellectual disability who is unable to express their will shall participate in scientific research’. Again, exceptions were not considered, disagreeing with the international standards mentioned above and leaving individuals with dementia excluded from the possibility of participating in scientific projects. Consequently, this negatively impacted dementia research in the country, further widening the existing gap. A recently published comprehensive data analysis showed that after Law 20.584 came into force in 2012 in Chile, there was a redistribution of the types of pathologies studied in the country. Specifically, the percentage of clinical trials addressing mental disorders decreased from 4 to 1% over the following 3 years.^59^

Almost a decade passed after this situation of discrimination against this vulnerable group of individuals was modified, when in 2021 the 21.331 law, which regulates the rights of individuals in mental healthcare, was issued.^60^ This law modified Article 28 of 20.584 law and currently states: ‘*Biomedical research cannot be conducted on adults who are physically or mentally incapable of expressing their consent or whose preference cannot be known, unless the physical or mental condition that prevents giving informed consent or expressing their preference is a necessary characteristic of the studied group’. ‘(…) the research protocol must contain specific reasons for including individuals with a condition that prevents them from expressing their consent or manifesting their preference. It must be demonstrated that the research involves a potential direct benefit for the individual and entails minimal risks to them. Furthermore, prior approval must be obtained from an accredited scientific ethics committee and authorization from the Regional Ministerial Health Secretariat. In those cases, the members of the committee evaluating the project must not be directly or indirectly affiliated with the center or institution where the research will be conducted, nor with the principal investigator or the project sponsor’.* ‘*(…) Individuals with neurodegenerative or psychiatric conditions may give their informed consent in advance to participate in future research trials, when they are not in a position to consent or express a preference (…)*’. The establishment of the 21.331 law represented an advancement of the Chilean legal system in the context of mental health and research in this area, providing a more detailed legal framework for the development of biomedical research with vulnerable groups including people with dementia. Yet, this law was not exempt from criticism from the academic community, which criticized, among other things, the lack of clarity in some clauses, as well as the law itself questioning the objectivity of the ethics committees.^57,61^ This highlights the intricate nature of the informed consent process, which can lead to the exclusion of individuals with dementia from biomedical research or clinical trials that could signify a direct benefit for them. Thus, it is crucial to advance towards legislation that not only protects people with dementia but also avoids discrimination and ensures their inclusion.

## Concluding remarks

AD research in LatAm presents both significant challenges and valuable opportunities. The region’s rapidly increasing prevalence of AD, coupled with unique social, cultural and economic factors, underscores the need for more inclusive and region-specific research. The underrepresentation of LatAm populations in global studies, combined with socioeconomic disparities and limited access to healthcare infrastructure, highlights the urgency of addressing these gaps. Despite these obstacles, LatAm’s diverse populations offer a rich source of data that can contribute to a broader understanding of the aetiology and progression of AD. Furthermore, ethical considerations in research, particularly in ensuring informed consent for individuals with cognitive impairment, remain paramount. The evolving legal frameworks, such as the recent changes in Chile, represent progress but also emphasize the need for continuous refinement to balance ethical integrity with the advancement of scientific knowledge.

## Data Availability

Data sharing is not applicable to this article as no new data were created or analysed in this study.
